# CpGAVAS, an integrated web server for the annotation, visualization, analysis, and GenBank submission of completely sequenced chloroplast genome sequences

**DOI:** 10.1186/1471-2164-13-715

**Published:** 2012-12-20

**Authors:** Chang Liu, Linchun Shi, Yingjie Zhu, Haimei Chen, Jianhui Zhang, Xiaohan Lin, Xiaojun Guan

**Affiliations:** 1 Institute of Medicinal Plant Development, Chinese Academy of Medical Sciences, Peking Union Medical College, 151 MaLianWa North Road, Haidian District, Beijing 100191, People’s Republic of China; 2 Center for Bioinformatics, University of North Carolina at Chapel Hill, Chapel Hill, NC, 27599, USA

**Keywords:** Chloroplast genome, Annotation, Web server, CPGAVAS

## Abstract

**Background:**

The complete sequences of chloroplast genomes provide wealthy information regarding the evolutionary history of species. With the advance of next-generation sequencing technology, the number of completely sequenced chloroplast genomes is expected to increase exponentially, powerful computational tools annotating the genome sequences are in urgent need.

**Results:**

We have developed a web server CPGAVAS. The server accepts a complete chloroplast genome sequence as input. First, it predicts protein-coding and rRNA genes based on the identification and mapping of the most similar, full-length protein, cDNA and rRNA sequences by integrating results from Blastx, Blastn, protein2genome and est2genome programs. Second, tRNA genes and inverted repeats (IR) are identified using tRNAscan, ARAGORN and vmatch respectively. Third, it calculates the summary statistics for the annotated genome. Fourth, it generates a circular map ready for publication. Fifth, it can create a Sequin file for GenBank submission. Last, it allows the extractions of protein and mRNA sequences for given list of genes and species. The annotation results in GFF3 format can be edited using any compatible annotation editing tools. The edited annotations can then be uploaded to CPGAVAS for update and re-analyses repeatedly. Using known chloroplast genome sequences as test set, we show that CPGAVAS performs comparably to another application DOGMA, while having several superior functionalities.

**Conclusions:**

CPGAVAS allows the semi-automatic and complete annotation of a chloroplast genome sequence, and the visualization, editing and analysis of the annotation results. It will become an indispensible tool for researchers studying chloroplast genomes. The software is freely accessible from http://www.herbalgenomics.org/cpgavas.

## Background

Regions on chloroplast genomes have been widely used as phylogenetic [1,2] and DNA barcoding markers [3-5] to determine the phylogenetic relationships of organisms and the identity of particular DNA samples. Furthermore, the complete sequences of chloroplast genomes provide important insights into the mechanism of molecular phylogeny and RNA editing, as well as the divergence of species [6-8]. With the rapid development of next-generation sequencing technology, the number of completely sequenced chloroplast genome is expected to increase exponentially [9-11]. Once the genome of a chloroplast has been assembled, accurate identification of genome features, such as genes coding for proteins, rRNA and tRNA, as well as inverted repeats, must be completed before additional analyses can be carried out. While the initial annotation can be performed with automatic annotation software, repeated manual editing by domain experts is required. A circular map is also needed to present various genomic features for visual inspection. Furthermore, the annotation results need to be submitted to GenBank for publication. Carrying out these steps can be tedious and time consuming for bench scientists. And they can easily become a bottle neck with the deluge of complete chloroplast genome sequences.

Protein-coding sequences (CDS) and exon-intron structures in genome sequences can be predicted either by *ab initio* predictions or sequence similarity methods. Several programs such as SNAP [12], Augustus [13], and Maker [14] have been widely used. Comparison of their performance showed that the sequence similarity approaches generally produce better results than *ab initio* gene prediction programs [15,16]. In terms of drawing circular chloroplast maps, several software packages and tools have been developed to suit this purpose [17-19]. While these tools can generate high-quality circular maps, they do not support interactive editing of the chromosomal features. Using these tools to generate circular maps will require repeated steps of updating the annotation details, generating the map, visualizing the map and inspecting the annotations to find errors. Alternatively, the domain experts can edit erroneous genomic features on the map off-line, using commercial graphic editing software tools such as Adobe Illustrator [17]. Both approaches are error-prone and tedious. In summary, an integrated software tool for the annotation of chloroplast genome is urgently needed to dealing with the deluge of chloroplast genome sequences.

Many command-line or web server versions of annotation pipelines have been developed for nuclear genomes. However, to our knowledge, there is only one web server, DOGMA, which is able to annotate chloroplast genomes specifically [20]. DOGMA has been extensively used and most chloroplast genomes currently available in GenBank were first annotated by DOGMA. However, our research group found several limitations in the use of DOGMA. First, the annotation pipeline of DOGMA is based on the local sequence similarity search tool Blastx [21], which is not suitable for defining the start and end of exons. Second, the editing function of DOGMA is not powerful comparing to modern annotation editing software tools such as Apollo. Third, DOGMA does not support the identification of inverted repeats. Forth, the output of DOGMA is not standard and requires reformatting for downstream data presentation or analyses, which can be a rather tedious step for experimental scientists. Last, DOGMA does not support the generation of circular maps, which are hallmarks of chloroplast genomes. In this study, we have developed a web server Chloroplast Genome Annotation, Visualization, Analysis, and GenBank Submission (CPGAVAS) in order to provide functions that support standard practices for annotating and analyzing chloroplast genome sequences, which are missing in DOGMA. CPGAVAS has several advantageous features, making it a potential turn-key solution for chloroplast genome annotation. It also can integrate the steps to manually edit the annotations using third-party tools easily. We hope CPGAVAS would relieve the bench scientists from the often tedious first tier annotation and analysis of Chloroplast genomes, and at the mean time, allow them to validate, edit and update the annotations and analysis results iteratively.

## Implementation

Chloroplast genome annotation can be divided into four tasks: (1) identifying protein coding genes, (2) identifying rRNA genes, (3) identifying tRNA genes, and (4) identifying inverted repeats. As described above, protein coding regions and exon-intron structures can be identified by *ab initio* gene prediction and similarity-based approaches. Chloroplast genomes are relatively small, with an approximate size between 120–160 kbp, and contain ~130 genes, which can be further divided into ~4 ribosomal RNA genes, ~30 transfer tRNA genes and ~80 protein coding genes. The methods that rely on the training of gene models for a given species are not applicable because of the lack of genes that can be used to train the models. As a result we developed our pipeline based on similarity-based methods.

**Figure 1:**
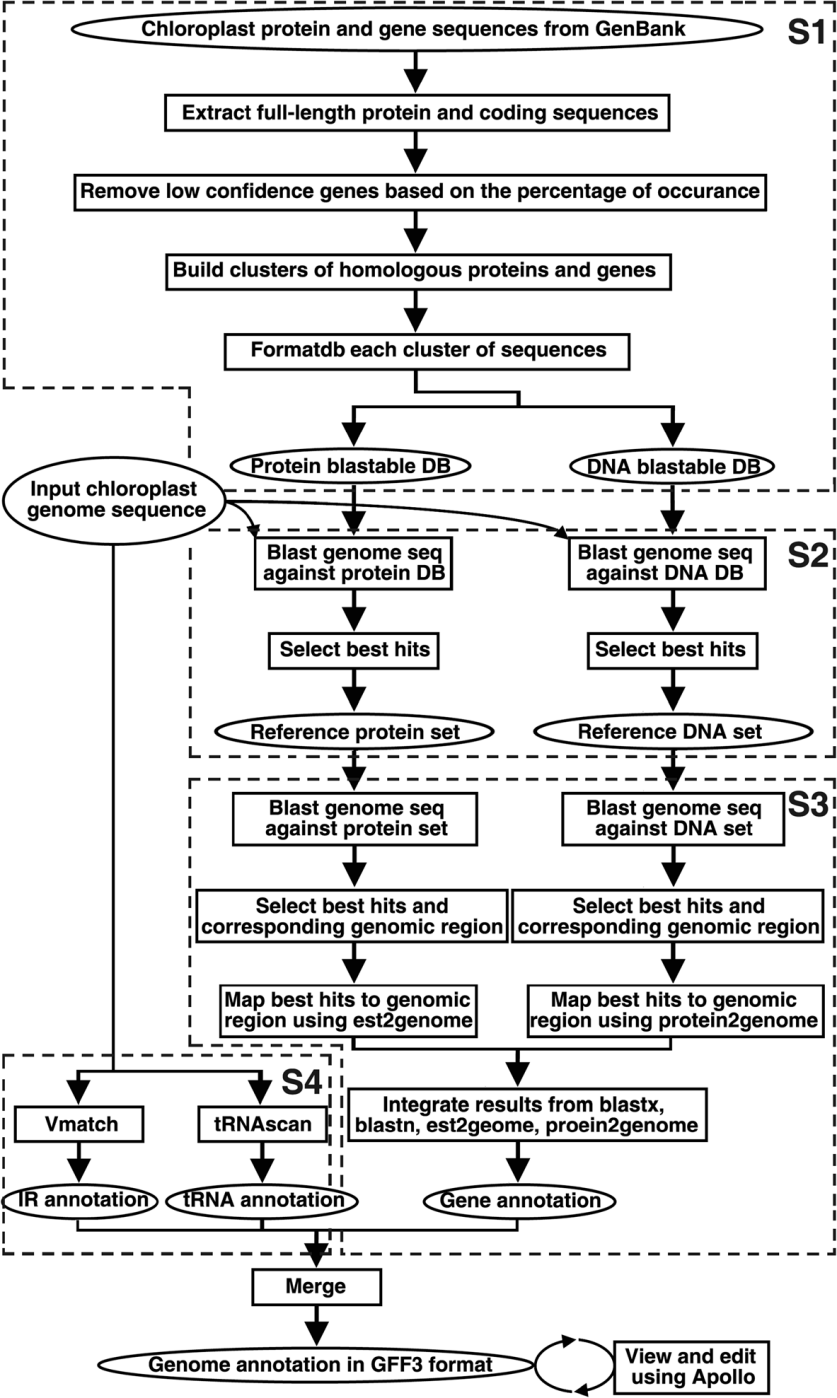
**Flow chart describing the CPGAVAS annotation pipeline.** The four steps (S1-S4) of the pipeline are shown in dashed frames.

The annotation pipeline of CPGAVAS is shown in Figure 1 and can be divided into four steps. In step 1, we cluster the protein, cDNA and “rRNA gene” sequences into homologous groups based on GenBank annotations and then create a blast-able database for each group. Briefly, we first extract all chloroplast protein, cDNA and “rRNA gene” sequences from GenBank. Only those records having a high level of confidence (the corresponding homologous groups having more than a specified number of members) are retained. Then, those predicted genes/proteins are removed. Homologous sequences for the remaining protein, cDNA, “rRNA gene” clusters are formatted into one blast-able database per group.

In step 2, we create a reference protein and a reference cDNA + “rRNA gene” dataset for each input query genome sequence. Briefly, the input genome sequence is searched against each cluster of protein, cDNA, “rRNA gene” blast-able databases created in step 1. A specified number of best hits from each cluster databases are extracted to build the corresponding reference protein and reference cDNA+”rRNA gene” dataset.

In step 3, the reference protein, cDNA and “rRNA gene” sequences are mapped to the genome sequence using Blastx, Blastn, protein2genome, and est2genome [22] programs. The results are then integrated as following. Each protein, cDNA and “rRNA gene” sequence in the reference dataset is called a hit. The regions on the genome having overlapping hits mapped to are merged to generate the “hit islands”. The “hit island” is used to group hits identified used the four different methods. Based on the number of different clusters of hits mapped to the same “hit island”, the “hit island” is broken into smaller “hit island”. Each of this “hit island” corresponds to a potential gene. For each “hit island”, the best full-length hit are selected and used to determine the structure of the corresponding gene using protein2genome or est2genome.

In step 4, the inverted repeats are identified using the vmatch software tool with default parameters [23]. And tRNAs are identified using tRNAscan [24] with the parameters specified by users. Because changing the parameter of intron length for tRNAscan can lead to significantly longer calculation time, we also predict the tRNA using ARAGORN, which has been shown to be able to recognize tRNA with introns in a reasonable amount of time [25]. In chloroplast genomes, the Met anticodon (CAU) is shared by trnI, trnfM and trnM, which can not be distinguished by tRNAscan. As a result, we construct three blast databases for coding sequences of trnI, trnfM and trnM respectively. The tRNAs recognized by tRNAscan as trnM are further compared to these three databases to determine if they are trnI, trnfM or trnM by Blast. Because of the relatively small size and the general lack of repetitive elements in chloroplast gnomes, we turn off RepeatMasker ( http://repeatmasker.org) in our pipeline. However the user has the option to turn it on.

**Figure 2:**
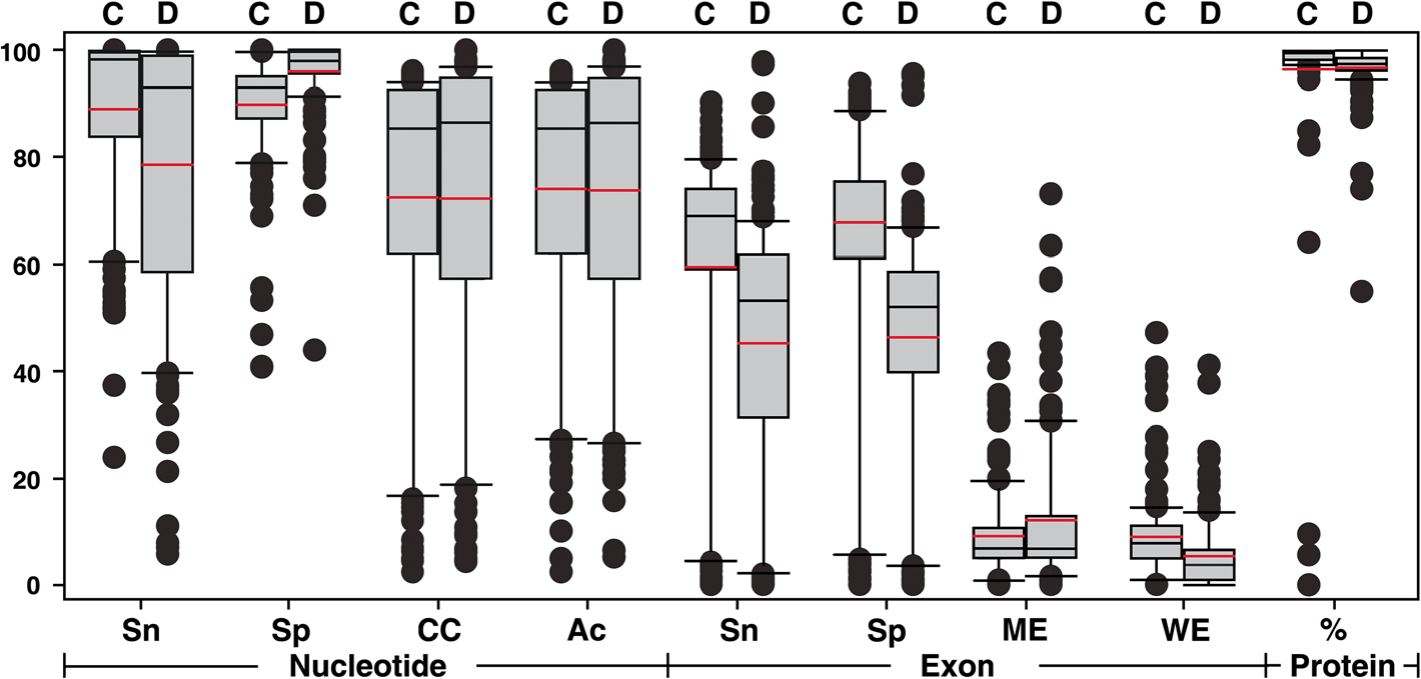
**Performance comparison of CPGAVAS and DOGMA annotation pipelines.** Box plots for the distributions of various parameters are shown. The mean is shown in red and the median is shown in black. The top and bottom of the box are the quartile lines. C: CPGAVAS; D: DOGMA; Sn: Sensitivity; Sp: Specificity; CC: Correlation Coefficient; Ac: Approximate Correlation; ME: Missing Exon; WE: Wrong Exon; PS: Percentage of Similarity.

To measure the performance of the CPGAVAS annotation pipeline, we retrieved 235 chloroplast genome records from GenBank and used GenBank’s annotations as true annotations, although GenBank’s annotations are known to contain errors. We then submitted these genome sequences to DOGMA and CPGAVAS for annotation. The measurement of annotation accuracy was carried out at three different levels: nucleotide, exon, and protein as described previously [26]. Basically, at the nucleotide level, we measured the accuracy of a prediction by comparing the predicted coding value (coding or non-coding) with the true coding value for each nucleotide along the test sequence. At the exon level, we compared the predicted and true exons to identify correct, wrong, and missing exons. At the protein level, we compared the predicted protein product with the true protein product and calculated the similarity score. It should be emphasized that we have excluded the query sequence itself from the reference database in the test. However, for DOGMA, we do not have access to the code and consequently can not exclude the query sequence from the backend database in the test. Overall, our CPGAVAS annotation tool showed a performance comparable to that of DOGMA (Figure 2). At the nucleotide level, it showed a better average sensitivity (0.9031 vs. 0.7339) and a slightly worse average specificity (90.65 vs. 95.16). In contrast, CPGAVAS showed a better average sensitivity (57.87 vs. 41.75) and specificity (50.09 vs 43.33) at exon level and better average percentage similarity (99.38 vs. 98.44) at protein level. The very poor annotation of a few species was due to the lack of reference sequences from closely related species. Our pipeline is actually similar to part of the Maker pipeline in terms of determining the gene structures. Maker’s performance has been shown to be equivalent or superior to several other leading annotation pipelines [14]. Consequently, performance comparisons between our CPGAVAS pipeline and those annotation tools are not repeated here.

The CPGAVAS web server was implemented using Perl Catalyst Web Application framework. The annotation pipeline was implemented in Perl programming language and calls the following external software tools: (1) Blastx and Blastn [21] to identify the full length proteins and cDNAs and rRNA genes that are most similar to a query sequence, (2) Blastx, Blastn, est2genome and protein2genome to map the most similar proteins, cDNAs and rRNA genes back to the query sequence, (3) tRNAScan and ARAGORN to identify tRNA, and (4) vmatch to identify the two inverted repeat elements. CPGAVAS is platform independent and has been successfully tested using various browsers, including Internet Explorer (7.0 and above), Mozilla Firefox (3.2 and above), and Opera, running under the Windows, Linux and MAC OS X operating systems. All scripts used in this study are available upon request.

## Results and discussion

### Input and output

The input is a chloroplast genome sequence in FASTA format. The output includes several files that contain: (1) annotation results in GFF3 format; (2) circular map of the annotated chloroplast genome in png format; (3) tables describing summary statistics of the genome; (4) annotation results combined with other user information in Sequin format. File 1 can be used to export the annotation results to any GFF3-compatible software tools, such as Chado [27], GBrowse [28], JBrowse [29], Apollo [30], and etc. for storage, presentation and editing. File 2 and 3 can be edited further for publication. File 4 can be used to submit the sequence to GenBank.

**Figure 3:**
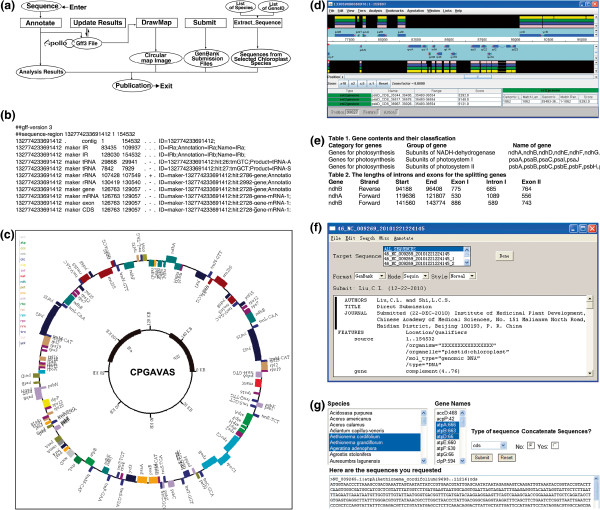
**The structure of CPGAVAS web server and its main results.** (**A**) The input, output, intermediate data (shown in eclipse) and the processes (shown in squares) for CPGAVAS; (**B**) A screen shot of the annotation results in GFF3 format; (**C**) a circular map of a chloroplast genome; (**D**) visualization and editing of the annotation results using Apollo software; (**E**) Summary statistics of CPGAVAS; (**F**) GenBank submission file seen in SeqIn application; (**G**) A screen shot of the input and output page for the “Extract Sequence” module.

An overall flowchart of the web server is shown in Figure 3A and each module of the web server is described in details below.

### Module 1: Annotate

This module is the core of this web server to provide automatic initial annotation and analysis of the genome of interests. The page allows users to submit their chloroplast genome sequence for analysis. Minimal information, such as the project and species names, is necessary to initiate an analysis. When users upload the sequence in FASTA format and submit a job, CPGAVAS will create a unique project id, by which users can retrieve the annotation results later. Modules 2–4, which are described next, facilitate the users to edit and update the annotation results, and re-calculate the genome statistics and re-draw the circular map accordingly.

### Module 2: ViewResults

This page takes a project id as an input, allowing users to retrieve all files associated with their annotation project. In addition to the annotation, map and report files, the users can download the sequences for the regions of predicted IR, rRNA gene, tRNA gene, protein coding gene, mRNA, CDS and protein.

### Module 3: UpdateResults

This page allows users to re-analyze the edited annotations using third party tools such as Apollo (Figure 3D). It takes the annotations described in GFF3 format (Figure 3B), re-draw the circular map (Figure 3C) and re-generate the analysis results (Figure 3E). Each update is given a unique id and the annotations can be retrieved from the “ViewResults” module later.

### Module 4: DrawMap

This page will take two different kinds of input files. One is the annotation results in GFF3 format (Figure 3B). This would allow the user to regenerate the circular map after editing the original annotations. Furthermore, it takes a file in a custom tab-delimited format, which can be generated easily use any text editors, given users maximal flexibility to draw a chloroplast circular map (Figure 3C).

### Module 5: Submit

The standard tools for submitting DNA sequences to GenBank include Sequin ( http://www.ncbi.nlm.nih.gov/Sequin/index.html) or BankIt ( http://www.ncbi.nlm.nih.gov/BankIt). This page organizes the GenBank sequence submission process into three simple steps, including: (1) providing contact and reference information by uploading the GenBank submission template file, (2) providing the sequence and its annotations by uploading FASTA and GFF3 files, and (3) providing sample information. After entering these information, two different files, one in Seqin format (Figure 3F) and another in GenBank format, will be generated and can be used for GenBank submission.

### Module 6: ExtractSeq

This page allows users to retrieve protein and mRNA sequences for lists of given genes and species name (Figure 3G). The sequences will be provided in two different formats, concatenated or non-concatenated, which can be subjected to phylogenetic analyses using either super-gene or super-tree methods [31].

We have not been able to improve the annotation accuracy to significantly exceed that of DOGMA. It seems to us that the computational tools are rather mature and the factors that would affect the prediction accuracy most is the availability of high quality reference sequences from closely related species. In addition, we found that different similarity cutoff (e.g., E values) will generate different annotation results. Consequently, users are suggested to try out different similarity cutoffs and then compare the results correspondingly. Ultimately, all predictions need experimental validation and manual correction of any errors is a must. This is why we have implemented CPGAVAS, which allows the annotation results to be visualized and edited using well developed third party software tools. Furthermore, CPGAVAS supports the re-processing of the edited annotations.

In the future, we aim to further refine the sequence extraction functions to allow the extraction of various sequence segments or segment combinations from one genome or multiple genomes belonging to particular taxonomy groups. These sequences will then be further pre-processed before they are subjected to alignment-based or alignment-free methods for phylogenetics, phylogenomics, and DNA barcoding studies.

## Conclusions

The rapid progress in next generation DNA sequencing technologies has already led to the deluge of completely sequenced genomes, particularly the small genomes such as those of the chloroplasts. Automatic, fast and integrated annotation and preliminary analysis of the complete genomes is a critical step connecting data generation and data interpretation. In this study, we have developed a complete pipeline that can annotate a chloroplast genome and perform preliminary analysis. In addition, it supports the manual curation of the automatic annotations using third party genome annotation software tools. We believe this tool would speed up the biological discovery based on sequencing and mining of the Chloroplast genomes.

## Availability and requirements

The software is freely accessible from http://www.herbalgenomics.org/cpgavas. As a web application, there is no requirement for the users to use the applications other than internet connections and browsers.

## Abbreviations

CpGAVAS: Chloroplast Genome Annotation, Visualization, Analysis, and GenBank Submission.

## Competing interests

The author(s) declare that they have no competing interests.

## Authors’ contributions

CL initiated the study. CL, LCS and YJZ implemented the web applications. CL wrote the manuscript. HMC, JHZ, XHL and XJG participated in the testing of the software. All authors have read and agreed with the contents of this manuscript.
